# Metastatic Extra Renal (Adrenal) TFE3 Translocation-Associated Renal Cell Carcinoma

**DOI:** 10.7759/cureus.75008

**Published:** 2024-12-03

**Authors:** Abdullah Alayed, Nayef Alshabyli, Saif Aldhali, Luay Alyamani, Fahad Alfarawi, Abdulmajeed Alfadhel

**Affiliations:** 1 Radiodiagnostics and Medical Imaging, Prince Sultan Military Medical City, Riyadh, SAU; 2 Radiodiagnostics and Medical Imaging, Prince Sultan Military Medical City, Riaydh, SAU; 3 Family and Community Medicine, Prince Sultan Military Medical City, Riyadh, SAU; 4 Pediatric Pulmonology, King Abdulaziz Medical City Riyadh, Riyadh, SAU

**Keywords:** adrenal tumors, extra renal carcinoma, metastatic renal cell carcinoma, oncogenic translocations, tfe3 translocation

## Abstract

Renal cell carcinoma (RCC) is the most common primary kidney neoplasm, with clear cell being the predominant histopathological type. Extra-renal RCC, a rare entity, occurs in locations outside the normal kidneys. This report presents a case of metastatic primary extra-renal RCC.

## Introduction

Renal cell carcinoma (RCC) is a common kidney malignancy, with clear cell carcinoma being the most prevalent histological type [1]. While RCC typically originates in the kidneys, extra-renal RCC is a rare condition, occurring in areas such as the retroperitoneum or adrenal glands, and metastatic RCC without an identifiable primary renal tumor is even more uncommon [2]. Primary extra-renal RCC in the pediatric population is extremely rare, with limited case reports available. This report describes a unique case of a 15-year-old female patient presenting with a left adrenal mass and metastatic lung lesion, despite no renal involvement, highlighting the diagnostic and therapeutic challenges associated with this rare form of RCC.

## Case presentation

**Figure 1:**
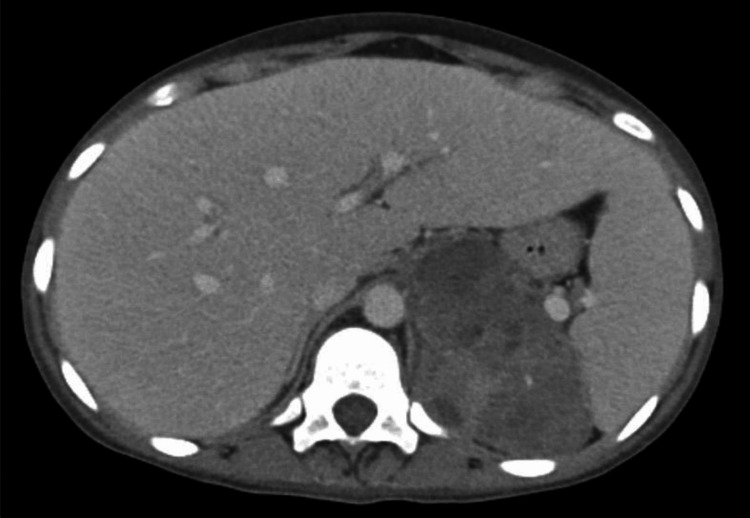
Axial enhanced CT scan showing a large heterogeneous left adrenal mass.

**Figure 2:**
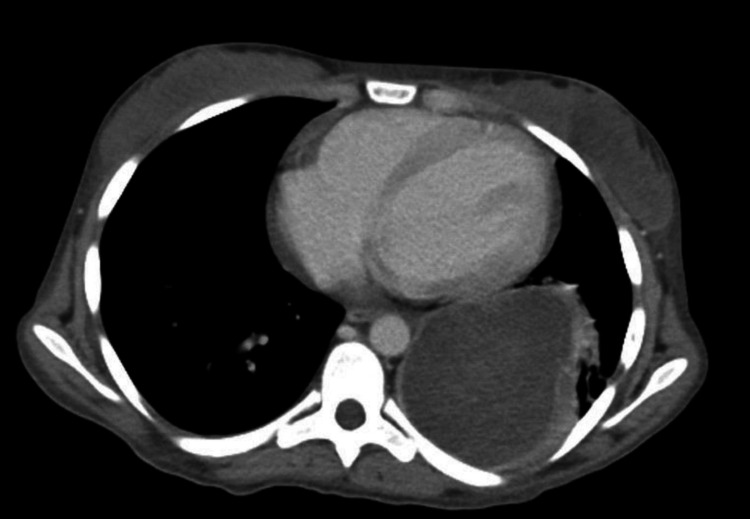
Axial enhanced CT scan showing a cystic lesion with atelectasis in the left lung.

**Figure 3:**
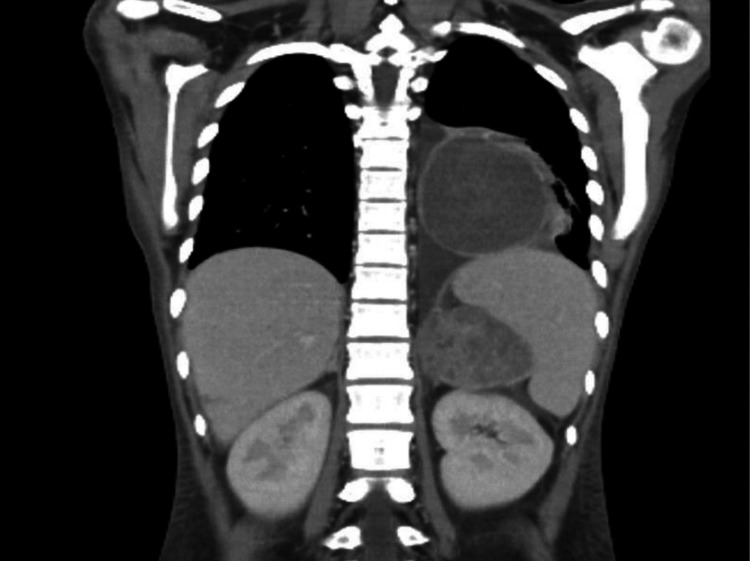
Coronal enhanced CT scan showing left adrenal and left lower lung lobe masses with unremarkable kidneys.

A 15-year-old female with no medical or surgical history presented with severe dizziness, fatigue, and weakness. Initial investigations included an abdominal ultrasound, which revealed a left adrenal mass. Staging CT of the chest, abdomen, and pelvis confirmed the adrenal mass and identified a left lung lesion (Figures 1-3), with no evidence of renal lesions. To determine the origin of the lesions, an ultrasound-guided percutaneous core needle biopsy of the adrenal mass was performed, with pathology suggesting MiT family translocation renal cell carcinoma.

Next, an open surgical biopsy of the left lung lesion confirmed metastatic carcinoma originating from the adrenal mass. The case was reviewed at a tumor board meeting, and due to the patient's respiratory failure requiring a high-flow nasal cannula from lung lesion compression, an initial left lower lung lobe resection was recommended. The surgery was performed, and pathology of the excised lobe demonstrated histological and immunohistochemical features consistent with MiT family translocation RCC.

Despite the absence of renal lesions, the patient was managed as a case of metastatic MiT translocation RCC with nivolumab, showing significant improvement. After eight months of systemic treatment, the patient was stable and underwent left adrenalectomy, with final pathology confirming an extra-renal (adrenal) TFE3 translocation-associated RCC (MiT family translocation RCC). The patient remained under active surveillance for one year, with no evidence of recurrence or metastasis.

## Discussion

To our knowledge, there are few reports of metastatic RCC without primary renal tumor. Only four of them reported retroperitoneal extra-renal RCC without renal lesions. Razi et al. reported a 78-year-old male with a left para-aortic lymph node mass and no identifiable renal masses, and after resection, the pathology revealed clear cell RCC. Fifteen months following the resection, there was no recurrent or metastatic disease [3]. Petrinec et al. have described the case of a 50-year-old female patient with a left para-renal mass who underwent left nephrectomy with resection of the para-renal mass; its pathology confirmed the diagnosis of translocation-associated RCC. Grossing of the mass precludes any connection of the mass with renal parenchyma, and preoperative imaging excluded any right renal lesions. The patient showed no evidence of disease recurrence on six months of follow-up by imaging [4]. Li et al. have published a case of a 63-year-old male who does not have any renal lesions; however, the pathology of the resected right retroperitoneal mass showed type 2 papillary RCC. Abdominal CT after five months of resection confirmed no recurrence [5]. Terada et al. have stated a case of an 81-year-old male patient with CT showing a right perinephric mass and no obvious renal masses. The mass was resected and pathologically proven to be separate from the adjacent renal parenchyma and consistent with extra-renal clear cell RCC [2]. Hasan et al. have reported the case of a 28-year-old female patient with a left adrenal mass demonstrated on CT without lesions in the kidneys or elsewhere in the body. After laparoscopic adrenalectomy, a pathological diagnosis of extra renal clear cell RCC was made. Six months of follow-up of the patient showed no evidence of recurrence [6]. 

All the cases were found to have a solitary primary extrarenal retroperitoneal RCC and remained disease-free on their follow-up after resection. Our case found similar findings; moreover, it is the only case proven to have metastatic disease. The reported cases were in an older age group, while our case is a primary extrarenal retroperitoneal RCC in the pediatric age group. Three of the published cases were diagnosed as clear cell type, one case of papillary type, and the last case is similar to our pathology diagnosis of translocation-associated type.

These rare cases of primary extra-renal RCC could be attributed to a few pathogeneses. One is that these tumors originate in ectopic renal cells from mesonephric remnants. Another possible explanation is the presence of occult renal primary, which is beyond the resolution of imaging. The last rationale is the reported tumors being metastatic from primary renal mass, which underwent spontaneous regression. 

## Conclusions

Primary extra-renal RCC is an exceptionally rare entity with several proposed explanations, including the development from ectopic renal cells originating from mesonephric remnants, the presence of an occult renal primary tumor undetectable by current imaging modalities, or metastasis from a primary renal tumor that has undergone spontaneous regression. These hypotheses reflect the diagnostic complexities of such cases and the rarity of their occurrence in clinical practice.

To the best of our knowledge, we present the first reported case of primary extra-renal RCC in the pediatric population with metastasis. This case uniquely demonstrates a favorable disease-free post-treatment course following comprehensive management. It underscores the importance of considering this rare diagnosis in unusual presentations and highlights the potential for effective therapeutic outcomes with timely intervention.
